# Opto-Thermoelectric Tweezers: Principles and Applications

**DOI:** 10.3389/fphy.2020.580014

**Published:** 2020-10-06

**Authors:** Agatian Pughazhendi, Zhihan Chen, Zilong Wu, Jingang Li, Yuebing Zheng

**Affiliations:** 1 Materials Science and Engineering Program and Texas Materials Institute, The University of Texas at Austin, Austin, TX, United States,; 2 Walker Department of Mechanical Engineering, The University of Texas at Austin, Austin, TX, United States

**Keywords:** optothermal effect, thermoelectricity, opto-thermoelectric tweezers, optical manipulation, colloidal particles

## Abstract

Opto-thermoelectric tweezers (OTET), which exploit the thermophoretic matter migration under a light-directed temperature field, present a new platform for manipulating colloidal particles with a wide range of materials, sizes, and shapes. Taking advantage of the entropically favorable photon-phonon conversion in light-absorbing materials and spatial separation of dissolved ions in electrolytes, OTET can manipulate the particles in a low-power and high-resolution fashion. In this mini-review, we summarize the concept, working principles, and applications of OTET. Recent developments of OTET in three-dimensional manipulation and parallel trapping of particles are discussed thoroughly. We further present their initial applications in particle filtration and biological studies. With their future development, OTET are expected to find a wide range of applications in life sciences, nanomedicine, colloidal sciences, photonics, and materials sciences.

## INTRODUCTION

Proposed by Ashkin [1, 2], optical tweezers have been broadly used to precisely manipulate bacteria, cells, quantum dots, plasmonic particles, and various dielectric particles in both two-dimensional (2D) and three-dimensional (3D) spaces [3–9]. Trapping colloidal particles at the center of the laser beam has found applications in nanomedicine, functional nanodevices, drug delivery, and fundamental studies [10–12]. Despite their wide applicability, conventional optical tweezers are difficult to achieve high spatial resolution in manipulation of particles at nanoscale due to the diffraction limit [2]. In addition, the common optical power density of conventional optical tweezers is in 10–10^3^ mW μm^−2^, for which fragile targeted objects such as gold nanoparticles and biological cells could be possibly damaged during the optical manipulation [13–16].

Various strategies were proposed to address these limitations of conventional optical tweezers [17–21]. For instance, plasmonic tweezers exploit the strong electromagnetic field enhancement in metallic nanostructures to achieve near-field trapping in plasmonic hotspots [22–24], which enable manipulation of nanoparticles and molecules beyond the diffraction limit with a reduced operational power [17]. Several techniques have been developed recently to cope with their spatially confined nature, leading to dynamical manipulation and long-range delivery of target objects based on plasmonic trapping [25–27]. Besides, optoelectronic tweezers can exploit light-induced virtual electrodes to generate dielectrophoretic forces for the manipulation of nanoparticles and living cells under a non-uniform electric field [28–30]. Electrothermoplasmonic flows have also been applied to work in conjunction with the localized plasmonic field to achieve long-range trapping of individual nano-objects [31, 32].

Recently, inspired by thermoelectric fields in ionic liquids due to electrolyte gradients [33], opto-thermoelectric tweezers (OTET) exploiting light-directed thermoelectric forces have been developed as a new type of optical manipulation technique. The dependence of thermoelectricity on temperature gradients instead of absolute temperature change allows OTET to operate at a significantly lower power compared to optical tweezers. In addition, the universal thermophoresis in the solutions makes OTET applicable to a wide range of polymers, metals, semiconductors, and dielectric nanostructures with different sizes and shapes. Furthermore, OTET can manipulate metal nanoparticles with a wide range of tunable working wavelengths, offering versatile platforms for *in situ* optical measurements where interference between manipulation and measurement beams can be minimized. By designing optothermal substrates with nanoscale heating sources, OTET can manipulate nanoparticles with spatial resolution beyond the diffraction limit [34]. This mini-review endeavors to summarize the working principles, recent developments, and applications of OTET. We start by discussing the optothermal effects and physical mechanisms involved in OTET. Next, we introduce the working principles of OTET, followed by recent advancements of OTET with focus on 3D trapping and parallel manipulation. Finally, we conclude with initial applications of OTET, particle filtration, and biological studies.

## MECHANISMS

OTET rely on the thermophoretic migration and the resultant spatial separation of different ions under light-induced temperature gradients in solutions. The operation of OTET and their applications involve two major physical phenomena, i.e., thermophoresis and thermoelectricity. In this section, we introduce the basic mechanisms of the opto-thermo-matter coupling in OTET.

### Thermophoresis

Thermophoresis, also known as the Soret effect, describes the directed movement of an object in response to a temperature gradient [35]. A wide variety of colloidal species in solution are subjected to the Soret effect. The drift velocities (*u)* of particles under the influence of a temperature gradient is given by

(1) $$u=-D_{T}\nabla T$$

where ∇*T* is the temperature gradient and *D*_*T*_ is the thermophoretic mobility.

Additionally, the steady-state concentration gradient profile is given by [36]

(2) $$\nabla c=-c\frac{D_{T}}{D}\nabla T=-cS_{T}\nabla T$$

where *c* is the concentration of the solutes, *D* is the Brownian diffusion coefficient, and *S*_*T*_ = *D*_*T*_/*D* is defined as the Soret coefficient. Since *D* of different components in a solution can vary with several orders of magnitude, the Soret coefficient enables a better description of the thermophoretic migration. Herein, *S*_*T*_ < 0 indicates thermophilic behavior while *S*_*T*_ > 0 implies the thermophobic motion.

### Thermoelectricity

When a temperature gradient is built in an electrolytic solution, ions migrate directionally due to the thermophoresis as introduced above. Depending on the Soret coefficients and other physical properties of the ions, such as the charge, solvation energy, and ionic radius, different ions in the solution will drift at different speeds. Thus, a steady spatial separation of ions with opposite charges will be formed, generating a thermoelectric field. This process is also known as the Seebeck effect [37]. The charged particles under this thermoelectric filed will move to either the cold or the hot region determined by their charge characteristics. In a steady state, the electric field created through the Seebeck effect under an imposed temperature gradient is given by [38–41]

(3) $$E_{T}=\frac{k_{B}T\nabla T}{e}\frac{\sum_{i}Z_{i}n_{i}S_{Ti}}{\sum_{i}Z_{i}^{2}n_{i}}$$

where *k*_*B*_ is the Boltzmann constant, *e* is the elemental charge, *T* is the ambient temperature, *i* represents the ionic species, and *n*_*i*_, *Z*_*i*_, *S*_*Ti*_ are the concentration, charge number, and Soret coefficient of the ionic species (*i*), respectively. The migration velocity and direction of the charged particles can be controlled by the type and concentration of electolytes in solution.

## DEVELOPMENT OF OPTO-THERMOELECTRIC TWEEZERS

OTET were first demonstrated for 2D manipulation of single metal nanoparticles on a thermoplasmonic substrate using extremely low optical power density (0.05–0.4 mW μm^−2^) [42]. After that, a series of work has been carried out to improve the throughput of manipulation and achieve particle manipulation in 3D. In this section, the fundamental working principle of OTET is first introduced, followed by discussion on recent improvements of OTET.

### Working Principle

**FIGURE 1 |:**
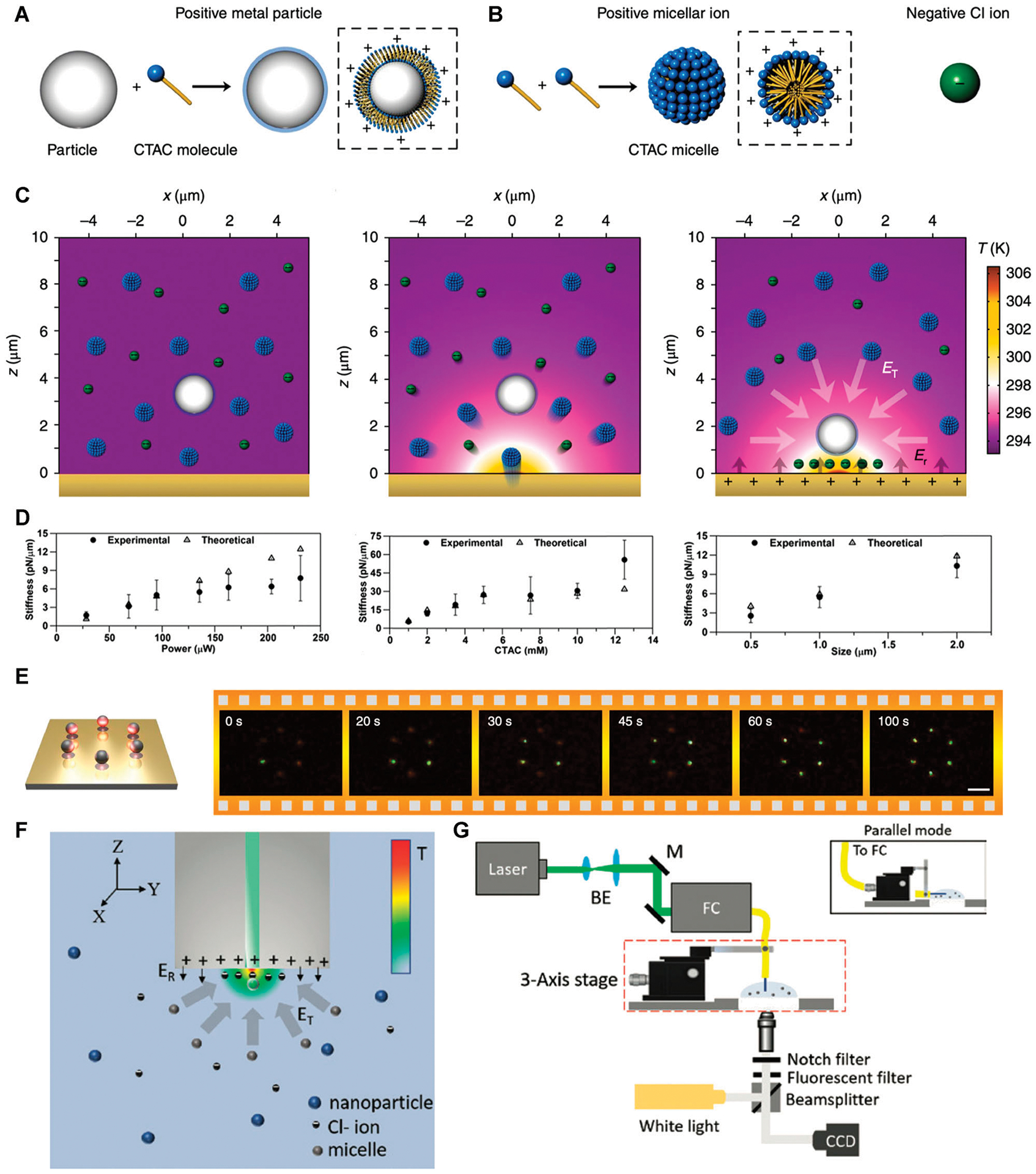
Working principles and recent developments of opto-thermoelectric tweezers (OTET). **(A)** Schematic of the surface charge modification of particles by cetyltrimethylammonium chloride (CTAC) adsorption. **(B)** Schematic of CTAC micelles and Cl^−^ ions. **(C)** Schematic of the working principle of OTET. **(D)** The trapping stiffness varies with the optical power (left), CTAC concentration (middle), and particle size (right). Adapted with permission from Ref. 46. Copyright 2019 American Chemical Society. **(E)** Parallel trapping of six Ag nanoparticles into a circular pattern by a digital micromirror device. **(A)**, **(B)**, **(C)** and **(E)** are adapted with permission from Ref. 42. Copyright 2018 Springer Nature. **(F)** Schematic of the mechanism and **(G)** the optical setup of opto-thermoelectric fiber tweezers. Adapted with permission from Ref. 48. Copyright 2019 De Gruyter.

Thermoelectricity has long been exploited to manipulate various colloidal particles [38], charged molecules [39], and micelles [40]. However, in previous works, certain electrolyte solution could only be applied to manipulate either positively or negatively charged particles, lacking the applicability to manipulate both types of charged particles simultaneously. In order to achieve more universal manipulation, Lin et al. [42] adopted cetyltrimethylammonium chloride (CTAC), a cationic surfactant, to enable the opto-thermoelectric trapping of various particles [43]. The CTAC molecules can be adsorbed onto the surface of colloidal particles regardless of their original surface charges, forming a positively charged molecular double layer (Figure 1A). Concurrently, the CTAC molecules cluster and self-assemble into micelles when the concentration is above the critical micelle concentration (0.13–0.16 mM) (Figure 1B). The CTAC micelles and the Cl^−^ ions can function as the counterions to facilitate the generation of the thermoelectric field for OTET.

In order to introduce a controllable source of optical heating and temperature gradients for the formation of the opto-thermoelectric field, a laser beam is directed onto a Au nano-islands (AuNIs) thermoplasmonic substrate, which is composed of quasi-continuous Au nanoparticles. The AuNIs substrate can convert the light into heat in a localized area near the laser spot. Upon laser heating of the AuNIs, both the CTAC micelles and Cl^−^ ions undergo thermophoretic migration from the hot to the cold region. However, since the CTAC micelles have a much larger Soret coefficient than that of Cl^−^ ions [S_T_ (micelle) ~10^–2^ K^−1^ >> S_T_ (Cl^−^) ~7.18 × 10^−4^ K^−1^], a stable spatial separation between them occurs and an electric field is formed with the direction pointing toward the hot region, i.e., the laser beam. The process of ion separation and subsequent production of an electric field, known as thermoelectricity, is a critical aspect of OTET, which separates it from other optothermal manipulations that rely purely on thermophoresis [44, 45]. The opto-thermoelectric force then traps the nanoparticles, which are positively charged either originally or due to surface modification by CTAC adsorption, around the hotspot on the substrate (Figure 1C).

Recently, Kollipara et al. [46] established a theoretical framework for the generation of the opto-thermoelectric field and resultant forces in complex colloidal systems to instruct the opto-thermoelectric manipulation of particles. This theoretical model considers the temperature variation and the sub-particle thermal conductivity variation caused by the trapped particles, which significantly improves the accurate calculation of thermoelectric forces. Upon the same laser irradiation with an optical power of ~135 μW, the theoretically calculated in-plane trapping potential of OTET for 1 μm polystyrene (PS) particle is roughly 400 k_B_T, which is 2–3 orders higher in magnitude than that of conventional optical tweezers [47]. Furthermore, the trapping stiffness of OTET was found to depend on three main factors: laser power, CTAC concentration, and the size of target particles (Figure 1D). Specifically, the trapping stiffness is predicted to linearly increase with an increase in laser power. However, the experimental results deviate from the simulated ones in the high-laser-power region because of the strong Rayleigh−Benard convection and Brownian motion at high temperatures. The trapping stiffness also increases with the CTAC concentration in the lower regime and then saturates. Accordingly, the concentration of CTAC is preferred at the onset of saturation for OTET to avoid the effect of strong depletion force. Besides, in a general OTET setup, when the particle size is less than 0.1 μm, the trapping stiffness has a square growth region with an increase in the particle size. However, when the size exceeds 0.5 μm, the trapping stiffness grows linearly with the particle size due to the synergistic effect of the surface charge and effective temperature gradient. A transition zone appears in the intermediate size regime.

### Improvement of Throughput

The manipulation throughput of OTET can be enhanced by trapping and manipulating multiple particles simultaneously.Two strategies have been developed: 1) using optical devices to generate multiple laser beams and 2) generating large-area opto-thermoelectric speckle fields to trap more particles.

A digital micromirror device (DMD) can split a single laser beam into multiple beams with programmable control over the beam size and shape. By programming the DMD, Lin et al. [42] have shown that multiple particles can be trapped simultaneously and transported along the designed paths (Figure 1E). Besides generating multiple laser beams for multiple manipulation, Kotnala et al. [49] exploited multiple-mode fibers to develop opto-thermoelectric speckle tweezers for large-scale opto-thermoelectric manipulation. The output of an excited multimode fiber leads to a speckle light pattern on the AuNIs substrate. The resultant large-area speckle light field can generate multiple thermoelectric hotspots to trap numerous nanoparticles simultaneously. The speckle pattern from a multimode fiber is advantageous as it has uniformly distributed high-intensity spots, high optical transmission efficiency, and simple alignment with the substrate.

### Three-Dimensional Manipulation

Despite larger trapping stiffness at low laser power, OTET on AuNIs is limited to 2D manipulation near the surface of the substrate. To further develop OTET as a multifunctional tool, 3D manipulation is an essential aspect.

Inspired by 3D manipulation based on optical fiber tweezers [50, 51], one practical method for 3D OTET is to transfer the optothermal substrate onto an optical fiber platform [48]. As shown in Figure 1F, the opto-thermoelectric fiber tweezers eliminate the need for traditional optical components such as mirrors and lenses in optical fiber tweezers, which makes it a simpler, alignment-free, and economical particle trapping technique. The primary feature of opto-thermoelectric fiber tweezers is the single-mode pigtail fiber (core/cladding equal to 9/125 μm) with a layer of AuNIs coated on the tip, which is affixed to a 3D-axis stage. The laser beam is channeled through the fiber with output power ~0.8 mW to create localized opto-thermoelectric fields at the tip (Figure 1G). Similarly, the positively charged particles can be trapped at the fiber tip due to the thermoelectric field. 3D manipulation can then be implemented by moving the 3D axis stage to control the position of the fiber tip. With 3D particle manipulation abilities, opto-thermoelectric fiber tweezers can function as nanopipettes for a wide range of applications in biosensing, additive manufacturing, and single nanoparticle-cell interactions.

## APPLICATIONS OF OPTO-THERMOELECTRIC TWEEZERS

OTET are promising for various applications due to their wide applicability to different particles, low operational power, and tunable working wavelengths. As initial demonstrations, particle filtration has been achieved via the synergistic effect of opto-thermoelectric force and Stokes drag force in microfluidic devices. In addition, the opto-thermoelectric fiber tweezers can be further developed into nanopipettes to precisely control the interactions between particles at nanoscale for biological studies.

### Particle Filtration

Size-based particle filtration is important in nanosciences. OTET-based particle filtration can be achieved by implementing opto-thermoelectric speckle tweezers in conjunction with microfluidic flows [49].

**FIGURE 2 |:**
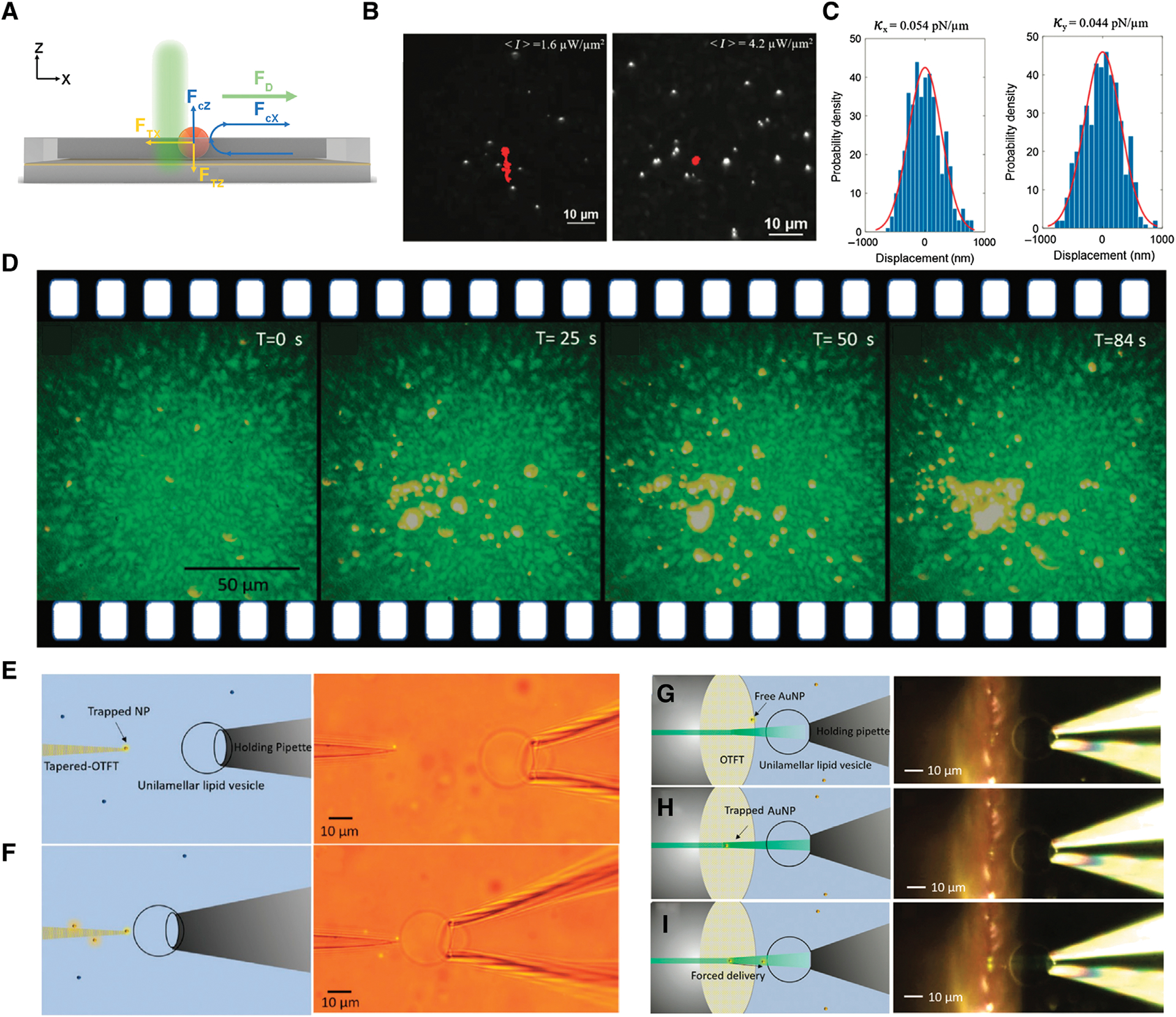
Particle filtration and nanopipettes based on opto-thermoelectric tweezers (OTET). **(A)** The schematic of the working mechanism of particle filtration. **(B)** Large-scale trapping of 500 nm PS beads with an average speckle intensity <*I*> 1.6 μW/μm^2^ (left) and <*I*> 4.2 μW/μm^2^ (right). The red lines are the trajectories of certain PS bead. **(C)** Position fluctuations of a single trapped 500 nm PS bead in the X (left) and Y (right) direction. **(D)** Time-lapsed optical images demonstrating that 200 nm PS beads can be filtered out from the mixture solution of 200 nm and 1 μm PS beads through opto-thermoelectric speckle tweezers and microfluidic flows. Adapted with permission from Ref. 49. Copyright 2020 De Gruyter. **(E)** Schematic (left) and optical image (right) showing that the tapered fiber traps the nanoparticle, ready for delivery. **(F)** Schematic (left) and optical image (right) demonstrating that a single 200 nm PS bead is directly delivered to the lipid vesicle. **(G)** Schematic (left) and optical image (right) showing the initial stage of the remote delivery. **(H)** Schematic (left) and optical image (right) showing the target nanoparticle being trapped on the fiber tip. **(I)** Schematic (left) and optical image (right) showing the target nanoparticle being delivered to the vesicle remotely by increasing the laser power. Adapted with permission from Ref. 48. Copyright 2019 De Gruyter.

As shown in Figure 2A, the trapping of a single particle by opto-thermoelectric speckle tweezers in a microfluidic channel is mainly affected by two types of forces - opto-thermoelectric force and Stokes drag force. The *F*_*Tx*_ and *F*_*Tz*_ represent the directional trapping forces created by the speckle opto-thermoelectric field that work in tandem to keep the particle at the hotspot. Figure 2B shows that multiple 500 nm PS beads are trapped under the opto-thermoelectric speckle field with laser power-dependent trapping stiffness (Figure 2C). The total Stokes drag force produced by the fluid flow is comprised of two components, i.e., the laminar drag force (*F*_d_) from the external microfluidic flow and the convective drag force (*F*_*cz*_ and *F*_*cx*_) from the localized natural convective flow.It should be noted that the localized convective flow causes a change in the direction of *F*_*cx*_ depending on the location of the particle relative to the Z-axis. As shown in Figure 2D, the particle-filtration system initially contained 200 nm and 1 μm PS beads with a microfluidic flow at velocity of 20 μm/s. At a selected speckle intensity, the drag force from the localized convection flow increased and *F*_*cz*_ pushed the 1 μm beads away from the substrate, causing *F*_*cx*_ dominating over *F*_*Tx*_. Thus, the 1 μm beads were carried away by the input microfluidic flow. In contrast, the 200 nm PS beads stayed trapped because the thermoelectric trapping force on these particles remained dominant over drag forces. Consequently, only the smaller particles were filtered out of the fluid solution, which differs from filtration based on optical speckle tweezers [52] because OTET-based filtration allows the larger particles to flow through the fluid channel while only retaining the smaller particles. The target size of the filtered particles can be changed by adjusting the speckle intensity, CTAC concentrations, and fluid flow velocities.

### Nanopipettes

Opto-thermoelectric fiber tweezers can be applied as a new type of nanopipettes to deliver objects and investigate the interaction between two arbitrary micro-/nano-objects with low operational power and high precision [48]. With a tapered fiber tip, opto-thermoelectric fiber tweezers can stably trap a particle at the tip with high accuracy and directly deliver the particle to another object (Figures 2E). For instance, a 200 nm PS nanoparticle was trapped at the tip and aligned with the lipid vesicle. Once aligned, the fiber was precisely moved to the vesicle until the nanoparticle was in contact with the surface of the vesicle (Figures 2F). Due to the low operational power, this technique shows special advantages in biological applications where the biomolecules have to be in the vicinity of the cell membrane for an extended period of time but without sacrificing the biological activity of the biomolecules. Moreover, remote delivery of nanoparticles based on opto-thermoelectric fiber tweezers can also be achieved. The target nanoparticle was still first trapped on the fiber tip and aligned with the vesicle (Figures 2G,H). The power output was then increased for a short duration to shoot the nanoparticle onto the vesicle by the strong optical scattering force (Figure 2I). The remote delivery via opto-thermoelectric fiber tweezers can transport nanoparticles at a distance of >10 μm with a good directional control, which prevents the potential physical damage from direct contact between the fiber tip and the object.

## SUMMARY

Optothermal manipulation of colloidal particles under light-directed thermoelectric fields has been demonstrated as an effective technique to trap and manipulate particles with a wide range of sizes, geometries, and compositions [53, 54]. Integrated with the optical fibers, OTET can improve the manipulation throughput and achieve 3D particle manipulation. Due to the low operational power and tunable working wavelengths, OTET are promising for a wide range of applications in nanomanufacturing, colloidal sciences, nanophotonics and life sciences. At present stage, OTET is limited by its lack of biocompatibility and manipulation efficiency, which can be further improved in the following three aspects: the type of electrolyte solutions, heating sources and intelligent manipulations.

The electrolyte solution of present OTET is composed of CTAC as active ions, which can facilitate both the formation of thermoelectric fields and the surface charge modification of colloidal particles. In the future, more types of applicable electrolytes are supposed to be discovered to fit in with different manipulation scenarios. For instance, CTAC may be harmful to some biological cells. Considering that most of the biological cells have negatively charged surfaces, to exploit OTET for some biological applications, biocompatible electrolytes should be found to enable biological and biomedical studies and applications.

The critical factor in increasing the performance of OTET is the optimization of light-controlled temperature gradients. In addition to AuNIs, the heating sources can be any other pre-designed patterns or nanostructures with high optothermal conversion efficiency and low thermal conductivity to build a localized temperature field. For example, a Si metasurface can be designed as the thermal substrate for OTET, which can be integrated with electronic devices for some biosensing applications [55]. Alternatively, the design of some mobile light-absorbing nanostructures such as Janus particles can not only help OTET eliminate the need for the thermal substrates but also add more functionalities to the manipulated objects.

Intelligent manipulation based on OTET is also highly desired to enhance the precision and throughput of optothermal assembly [56, 57]. Several types of optical devices, such as DMDs [58], spatial light modulators [59, 60] and acousto-optical deflectors [61], have been proved promising in parallel manipulation of multiple colloidal particles. All these devices can be controlled automatically via homebuilt programs to manipulate multiple particles simultaneously along the pre-designed paths. Moreover, for 3D optothermal assembly, the out-of-plane manipulation can be very sensitive to the laser focal plane. Thus, imaging process integrated into the laser-controlled programs is necessary to achieve feedback control for automated optothermal assembly. With the versatile manipulation and assembly capabilities, the intelligent manipulation based on OTET is promising in efficient and high-throughput fabrication of a wide range of functional devices such as colloidal waveguides, metasurfaces, photonic crystals and so forth.
